# Down-regulation of microRNA-203-3p initiates type 2 pathology during schistosome infection via elevation of interleukin-33

**DOI:** 10.1371/journal.ppat.1006957

**Published:** 2018-03-19

**Authors:** Xing He, Jun Xie, Yange Wang, Xiaobin Fan, Qin Su, Yue Sun, Nanhang Lei, Dongmei Zhang, Guangping Gao, Weiqing Pan

**Affiliations:** 1 Department of Tropical Infectious Diseases, Second Military Medical University, Shanghai, China; 2 Horae Gene Therapy Center, University of Massachusetts Medical School, Worcester, Massachusetts, United States of America; 3 Department of Microbiology and Physiological Systems, University of Massachusetts Medical School, Worcester, Massachusetts, United States of America; New York University, UNITED STATES

## Abstract

The type 2 immune response is the central mechanism of disease progression in schistosomiasis, but the signals that induce it after infection remain elusive. Aberrant microRNA (miRNA) expression is a hallmark of human diseases including schistosomiasis, and targeting the deregulated miRNA can mitigate disease outcomes. Here, we demonstrate that efficient and sustained elevation of miR-203-3p in liver tissues, using the highly hepatotropic recombinant adeno-associated virus serotype 8 (rAAV8), protects mice against lethal schistosome infection by alleviating hepatic fibrosis. We show that miR-203-3p targets interleukin-33 (IL-33), an inducer of type 2 immunity, in hepatic stellate cells to regulate the expansion and IL-13 production of hepatic group 2 innate lymphoid cells during infection. Our study highlights the potential of rAAV8-mediated miR-203-3p elevation as a therapeutic intervention for fibrotic diseases.

## Author summary

Schistosomiasis is a serious but neglected tropical infectious disease. Hepatic fibrosis caused by lodged eggs from the parasite is the primary cause of morbidity and mortality from this disease. Type 2 immune response, featured by the T helper 2 cell associated cytokines such as IL-4 and IL-13, is the central regulator of disease progression in schistosomiasis, but the signals that induce it after infection remain elusive. Aberrant expression of miRNAs underlies a spectrum of human diseases, including infectious diseases. In this study, using a well-studied murine model of human schistosomiasis, we show that *Schistosoma* infection down-regulates the miR-203-3p expression, and that IL-33, an inducer of type 2 immunity, is a direct target of miR-203-3p in hepatic stellate cells. The reduced miR-203-3p leads to elevated levels of IL-33, promoting the expansion and IL-13 production of hepatic group 2 innate lymphoid cells and thus initiating type 2 pathology. Importantly, rAAV8-mediated elevation of miR-203-3p in liver tissues protects mice against lethal schistosome infection by alleviating hepatic fibrosis. Thus, our study highlights the crucial role of miR-203-3p in the initiation of type 2 pathology during schistosome infection, and suggests miR-203-3p as a potential target for fibrotic diseases.

## Introduction

Schistosomiasis is a serious but neglected tropical infectious disease, affecting more than 230 million people worldwide [1]. Hepatic granuloma and secondary fibrosis caused by lodged eggs from the parasite are the primary cause of morbidity and mortality from this chronic and debilitating disease. Elucidating the mechanisms that initiate hepatic schistosomiasis has been a major research objective for decades, and it is now well-established that hepatic schistosomiasis is an immune pathological disease [2,3]. A major breakthrough was the identification of type 2 immune response, characterized by the T helper 2 cell associated cytokines such as interleukin 4 (IL-4) and IL-13, as a central regulator of disease progression in schistosomiasis [2,3]. However, the signals that induce type 2 immune response after infection remain elusive.

Quiescent hepatic stellate cells (HSCs) are located in the subendothelial space, between the anti-luminal side of sinusoidal endothelial cells and the basolateral surface of hepatocytes, and are characterized by their cytoplasmic vitamin droplets [4]. When liver injury occurs, quiescent HSCs are activated to become proliferative, contractile, and fibrogenic myofibroblasts [5]. Activated HSCs produce excessive extracellular matrix (ECM) that is deposited in the liver, and are the main effector cells in various types of hepatic fibrosis, including fibrosis induced by schistosome infection [6]. In addition, more recent studies have uncovered the fundamental role of HSCs in hepatic inflammation and immunity [7,8].

MicroRNAs (miRNAs) are endogenous, small noncoding RNAs which control the activity of more than 30% of protein-coding genes through target mRNA degradation or translational inhibition [9,10]. Increasing evidence has demonstrated that miRNAs are involved in regulating almost every cellular process, and aberrant miRNA expression is a hallmark of many human disorders, including infectious diseases [11,12]. Several studies by ours and other groups have shown that miRNAs play a crucial role in the pathogenesis of schistosomiasis and may serve as useful therapeutic targets [13–15]. In particular, one of our previous studies has shown that depletion of a single miRNA, miR-21, in the liver protects hosts from lethal infection through attenuation of hepatic fibrosis [15].

In this study, we used a murine model of *Schistosoma japonicum* (*S*. *japonicum*) to investigate the role of miR-203-3p, a miRNA down-regulated following infection in the progression of hepatic schistosomiasis [15]. We found that recombinant adeno-associated virus 8 (rAAV8) mediated elevation of miR-203-3p in the liver protected mice from lethal infection through alleviating type 2 pathology. Importantly, our data indicate that miR-203-3p targets IL-33, an inducer of type 2 immunity [16,17], in HSCs to regulate the expression of IL-13 in hepatic group 2 innate lymphoid cells (ILC2s) during infection.

## Results

### rAAV8-mediated elevation of miR-203-3p protects mice against lethal schistosome infection by attenuation of type 2 pathology

**Fig 1:**
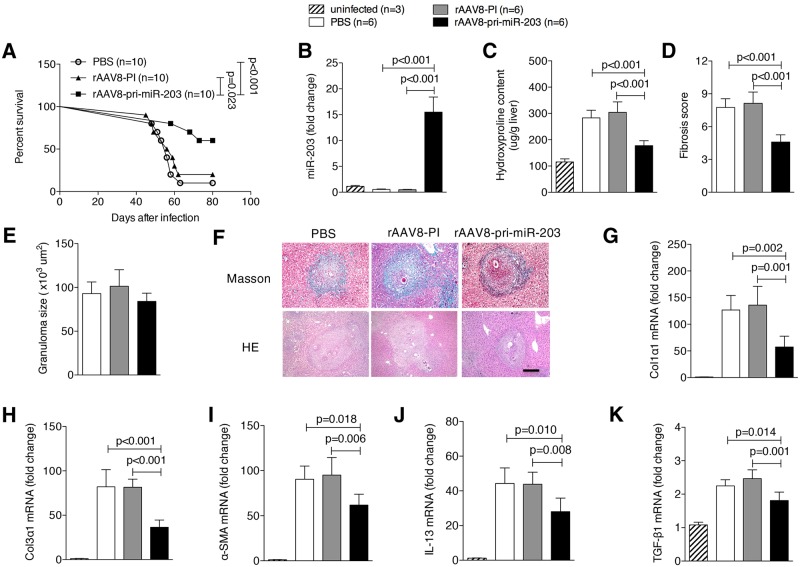
Elevation of miR-203-3p protects hosts from lethal schistosome infection by relieving type 2 pathology. (A) Mice were infected percutaneously with 30 *S*. *japonicum* cercariae at day 0 and treated with rAAV8-PI (n = 10) or rAAV8-pri-miR-203-3p vectors (n = 10) at a dose of 1×10^11^ virus genomes, or PBS (n = 10) by tail vein injection at day 10 post-infection. The animals were subjected to an 80-day survival study. Data are representative for three independent experiments. Survival between infected groups was compared by Kaplan–Meier survival curves with log-rank test. (B-K) Mice were infected percutaneously with 16 *S*. *japonicum* cercariae at day 0 or remained uninfected. Infected mice received rAAV8-PI or rAAV8-pri-miR-203-3p vectors at a dose of 1×10^11^ virus genomes or PBS by tail vein injection at day 10 post-infection. Liver samples were collected at day 42 post-infection. qPCR analysis of levels of miR-203-3p in the liver samples (B). Striped bars, uninfected mice (n = 3); white bars, mice receiving PBS (n = 6); grey bars, mice receiving rAAV8-PI (n = 6); black bars, mice receiving rAAV8-pri-miR-203-3p (n = 6). Collagen content in the livers determined as hydroxyproline content (C). Fibrosis scores measured from Masson’s trichrome staining of liver sections (D). Granuloma size measured from H&E staining of liver sections (E). Masson’s trichrome staining and H&E staining of liver sections (F). Scale bar, 200 μm. qPCR analysis of mRNA levels of collagen 1 (G), collagen 3 (H), *α-Sma* (I), *Il13* (J), and *Tgf-β1* (K). Data are expressed as the mean ± s.d. from two independent experiments. Multiple comparisons were performed by one-way ANOVA, and followed by Bonferroni post test for comparison between two groups.

We previously identified more than thirty deregulated host miRNAs by expression profiling during the progression of hepatic schistosomiasis. This includes miR-203-3p as the most down-regulated [15]. To examine the role of miR-203-3p in schistosomiasis *in vivo*, mice were first challenged with a lethal dose of *S*. *japonicum* cercaria and then intravenously injected with either rAAV8-pri-miR-203-3p vector sustainedly expressing the miRNA, control vector, or PBS at day 10 post-infection. We found that a single dose of rAAV8-pri-miR-203-3p protected infected mice from the lethal effect of schistosomiasis. Six of ten mice receiving rAAV8-pri-miR-203-3p survived to the end of the study (i.e. 80 days; Fig 1A). In contrast, the majority of mice receiving control vector (n = 10) or PBS (n = 10) died within 9 weeks post-infection (Fig 1A).

Hepatic granuloma and fibrosis, induced by host type 2 immune response resulting from liver-trapped parasite eggs, are the primary cause of morbidity and mortality from this disease [2,3]. Thus, we next investigated if the rAAV8-pri-miR-203-3p-mediated intervention was indeed through effective elevation of miR-203-3p activity that in turn attenuated the type 2 pathology. To this end, mice were exposed to a mild dose of parasites and then treated with vectors or PBS. Our data revealed that the level of miR-203-3p in the rAAV8-pri-miR-203-3p treated group was significantly higher than in the control groups (Fig 1B). Excessive ECM deposition is the main feature of hepatic fibrosis. By 6 weeks post-infection, mice receiving rAAV8-pri-miR-203-3p displayed a significant reduction in ECM deposition as shown by hydroxyproline quantification (Fig 1C) and Masson’s trichrome staining (Fig 1D and 1F), whereas the size of hepatic granulomas in all groups was similar as shown by H&E staining (Fig 1E and 1F). Reduction of fibrosis was further confirmed by qPCR-based quantification of fibrosis associated gene expression in the livers of infected mice. Amounts of *Col1α1*, *Col3α1*, and *α-Sma* mRNA were dramatically reduced in livers of mice treated with rAAV8-pri-miR-203-3p (Fig 1G, 1H and 1I). In addition, we detected the alteration of the cytokines that are associated with type 2 immune response in liver tissues after elevation of miR-203-3p. Consistent with the antenuated fibrotic phenotype, a strong reduction in mRNA levels of *Il13* and *Tgf-β1* was detected in livers of mice treated with rAAV8-pri-miR-203-3p (Fig 1J and 1K). However, levels of other cytokines, including interferon-γ (*Ifn-γ*), tumor necrosis factor-α (*Tnf-α*), *Il4*, and *Il5*, were not significantly altered (S1A Fig). Consistent with our previous study, the virus vector did not affect the survival and egg production of parasites in the hosts (S1B Fig), and virus delivery in the liver showed no significant differences between groups (S1C and S1D Fig).

HSCs are the predominant cellular source of ECM during hepatic fibrosis. Thus, we investigated whether the anti-fibrotic effect of rAAV8-pri-miR-203-3p intervention directly modulated the activity of HSCs. To this end, we isolated primary HSCs from infected mice after administration with vectors to quantify mRNA levels of miR-203-3p, *Col1α1*, *Col3α1*, and *α-Sma*. Our data showed that significantly decreased miR-203-3p expression and increased collagen and *α-Sma* expression were observed in the infected mice without rAAV8-pri-miR-203-3p treatment compared with uninfected mice (S2 Fig). As expected, miR-203-3p expression was clearly elevated, while collagen and *α-Sma* expressions were distinctly reduced in HSCs after rAAV8-pri-miR-203-3p intervention (S2 Fig), suggesting that miR-203-3p could modulate the activation of HSCs *in vivo*.

### rAAV8-mediated elevation of miR-203-3p has therapeutic potential for hepatic fibrosis induced by schistosome infection

**Fig 2:**
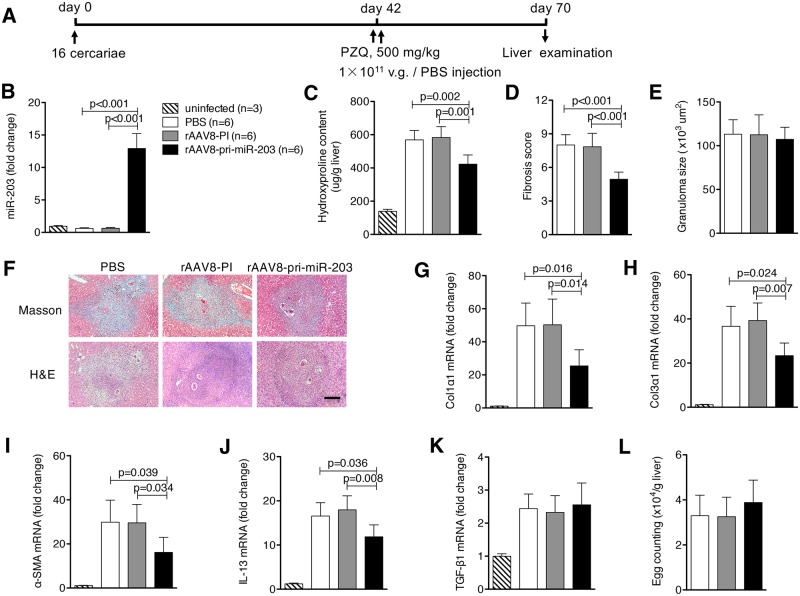
Elevation of miR-203-3p partially reverses schistosome-induced hepatic fibrosis. (A) Time schedule for parasite infection and administration of anti-parasite drug or virus vectors and sample withdrawal. Mice were infected percutaneously with 16 *S*. *japonicum* cercariae or remained uninfected. The infected mice were treated with praziquantel to kill the parasites, then received rAAV8-PI or rAAV8-pri-miR-203-3p vectors at a dose of 1×10^11^ virus genomes or PBS by tail vein infection at day 42 post-infection. Liver samples were collected at day 70 post-infection. (B) qPCR analysis of levels of miR-203-3p in the liver samples. Striped bars, uninfected mice (n = 3); white bars, mice receiving PBS (n = 6); grey bars, mice receiving rAAV8-PI (n = 6); black bars, mice receiving rAAV8-pri-miR-203-3p (n = 6). (C, D) Collagen content of the livers was measured by (C) hydroxyproline content or (D) fibrosis score determined from Masson’s trichrome staining of liver sections. (E) Granuloma size measured from H&E staining of liver sections. (F) Masson’s trichrome staining and H&E staining of liver sections. Scale bar, 200 μm. (G-K) qPCR analysis of mRNA levels of collagen 1 (G), collagen 3 (H), *α-Sma* (I), *Il13* (J), and *Tgf-β1* (K). (L) Egg burdens in the livers. Data are expressed as the mean ± s.d. from two independent experiments. Multiple comparisons were performed by one-way ANOVA, and followed by Bonferroni post test for comparison between two groups.

To investigate whether elevation of miR-203-3p in the liver can reverse the parasite egg-induced hepatic fibrosis, mice were infected with a mild dose of parasites. At 42 days after infection, when hepatic fibrosis was clearly manifest, mice were treated with praziquantel to kill the parasite, then injected with either rAAV8 vectors or PBS, and necropsied at 70 days post-infection (Fig 2A). Again, the expression of miR-203-3p was significantly increased in the rAAV8-pri-miR-203-3p treated mice (Fig 2B). Importantly, hydroxyproline quantification and Masson’s trichrome staining revealed that fibrosis in rAAV8-pri-miR-203-3p treated mice was markedly reduced compared with controls (Fig 2C, 2D and 2F), but the size of hepatic granulomas in all groups was similar as shown by H&E staining (Fig 2E and 2F). This was confirmed by reduced expression of *Col1α1*, *Col3α1* and *α-Sma* in these mice (Fig 2G, 2H and 2I). Of cytokines tested, only *Il13* mRNA was reduced (Fig 2J and 2K), and livers showed no significant change in egg burden (Fig 2L).

### IL-33 is a direct target of miR-203-3p in HSCs

**Fig 3:**
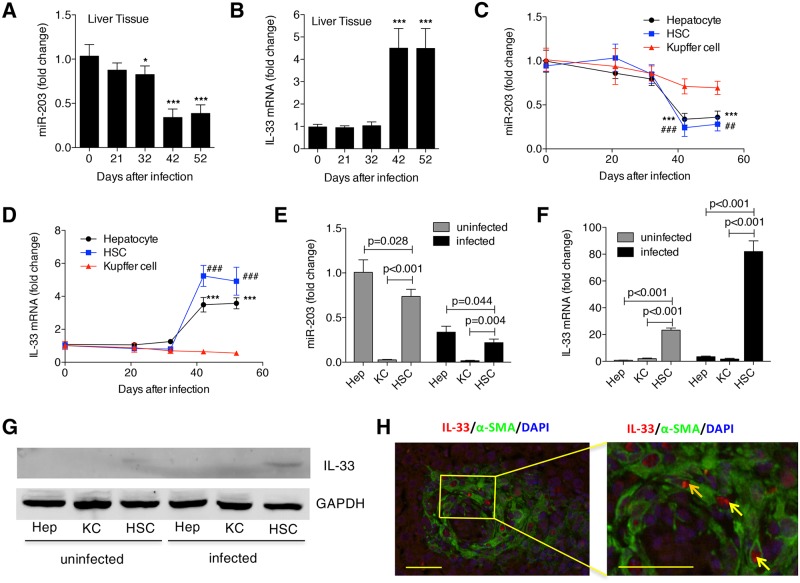
Analysis of miR-203-3p and IL-33 expression in the liver during infection. (A, B) The expression of miR-203-3p (A) and *Il-33* mRNA (B) in the livers during infection was detected by qPCR. * *P*<0.05, *** *P*<0.001, compared with samples of day 0. (C, D) The expression of miR-203-3p (C) and *Il-33* mRNA (D) in different types of primary liver cells during infection was detected by qPCR. *** or ### *P*<0.001, compared with samples of day 0. (E, F) Primary hepatocyte, HSCs, and Kupffer cells (KCs) were isolated from uninfected and infected (day 42 post-infection) livers, and the levels of miR-203-3p (E) or *Il33* mRNA (F) in HSCs and KCs were compared to those in hepatocytes from uninfected livers. (G) The expression of IL-33 in different types of primary liver cells from uninfected mice or infected (day 42 post-infection) mice was detected by western blot. (H) IL-33 (red) colocalization with α-SMA (green) in the infected (day 42 post-infection) liver sections was detected by immunohistochemistry. Cells staining positive for both IL-33 and α-SMA are indicated with arrows. The right picture is an amplified part of the left picture. Data are expressed as the mean ± s.d. from three independent experiments. Multiple comparisons were performed by one-way ANOVA, and followed by Bonferroni post test for comparison between two groups.

Considering that elevation of miR-203-3p attenuates type 2 pathology, we speculated that miR-203-3p could regulate the initiation of type 2 immunity after infection. IL-33, an IL-1-related cytokine, is an inducer of type 2 immunity in several organs [18], and is a potential target of miR-203-3p, predicted by TargetScan database. To analyze the relationship between miR-203-3p and IL-33, we evaluated their expression during the progression of hepatic schistosomiasis. We found that expression of miR-203-3p began to decrease in the liver at day 32 post-infection, reaching its lowest levels at day 42 and 52 (Fig 3A); in contrast, the level of *Il33* mRNA was maintained during the early stage of infection, then significantly elevated by day 42 post-infection (Fig 3B). In addition, we investigated the expression of miR-203-3p and *Il33* mRNA in different hepatic cell compartments, including hepatocytes, HSCs, and Kupffer cells (KCs). Our data showed that, similar to the expression pattern in whole liver, the expression of miR-203-3p in hepatocytes and HSCs began to decrease at day 42 post-infection, while the level of *Il33* mRNA was elevated at the same time (Fig 3C and 3D). However, the expression of both miR-203-3p and *Il33* mRNA in KCs was unchanged during the observed time (Fig 3C and 3D). We also characterized the relative abundance of miR-203-3p and *Il33* mRNA in different hepatic cell compartments, and found that, in both the uninfected and infected livers, miR-203-3p was selectively expressed in hepatocytes and HSCs (Fig 3E), whereas *Il33* was primarily expressed in HSCs (Fig 3F and 3G). To further validate that activated HSCs could be a source of IL-33 in infected livers, we carried out immunochemistry staining for IL-33 and α-SMA, and we observed that both factors were mainly located in the periphery of egg granulomas (S3 Fig). Double staining using immunofluorescence displayed a co-localization of IL-33 and α-SMA staining (Fig 3H), indicating that activated HSCs express IL-33 *in vivo*. In addition, we detected the expression of miR-203-3p and *Il33* during the progression of HSC activation *in vitro*. Resting HSCs will be automatically activated when cultured on a plastic surface [4]. We found that, when primary HSCs from uninfected mice were cultured on plastic plates, expression of miR-203-3p in these cells was significantly reduced, while the level of *Il33* mRNA was significantly elevated, accompanied by a dramatic increase in collagen expression (S4 Fig). Taken together, these results suggest that the activated HSCs in infected livers are a source of IL-33, and that IL-33 is a potential target of miR-203-3p in HSCs.

**Fig 4:**
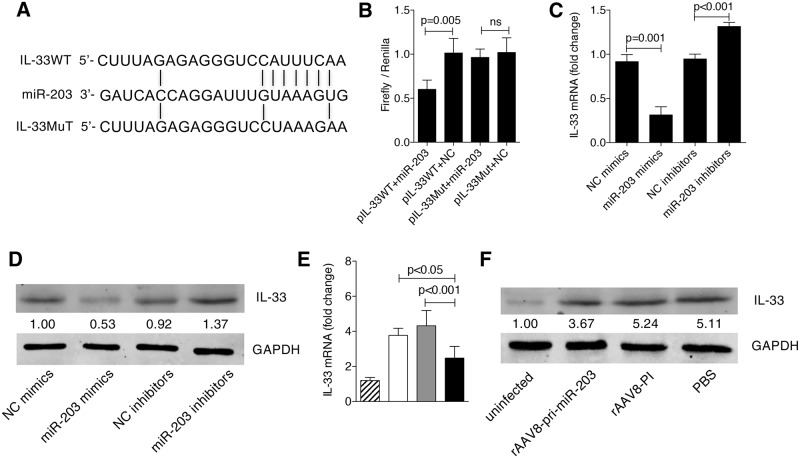
Validation of the relationship between miR-203-3p and IL-33. (A) Sequence alignment of miR-203-3p and its target sites in 3’ UTRs of *Il33*. (B) Luciferase reporter assays for 293T cells transfected with pRL-TK vectors carrying *Il33* wild type (WT) 3’ UTR or *Il33* mutant (Mut) 3’ UTR in the absence or presence of miR-203-3p mimics. (C, D) Primary HSCs were isolated from uninfected mice and cultured on a plastic plate. After 3 days in culture, cells were transfected with 40 nM miR-203-3p mimics, negative control (NC) miRNA mimics, miR-203-3p inhibitors, or negative control (NC) miRNA inhibitors for 48 h, then the expression of IL-33 was detected by qPCR (C) and western blot (D). (E, F) Mice were infected percutaneously with 16 *S*. *japonicum* cercariae at day 0 or remained uninfected. Infected mice received rAAV8-PI or rAAV8-pri-miR-203-3p vectors at a dose of 1×10^11^ virus genomes or PBS by tail vein injection at day 10 post-infection. Primary HSCs were isolated at day 42 post-infection, then total RNA and protein were collected and analyzed for expression of IL-33 by qPCR (E) or western blot (F). Striped bars, uninfected mice (n = 3); white bars, mice receiving PBS (n = 3); grey bars, mice receiving rAAV8-PI (n = 3); black bars, mice receiving rAAV8-pri-miR-203-3p (n = 3). Data are expressed as the mean ± s.d. from three independent experiments. Multiple comparisons were performed by one-way ANOVA, and followed by Bonferroni post test for comparison between two groups.

To test whether IL-33 is a direct target of miR-203-3p, we first generated a reporter construct that contains the firefly luciferase gene fused to the 3’ UTR from *Il33* mRNA containing a putative miR-203-3p target site (Fig 4A). This construct was transiently transfected into 293T cells together with miR-203-3p mimics or a negative control miRNA. We observed a marked reduction in luciferase activity in cells transfected with miR-203-3p mimics together with *Il33*-UTR (Fig 4B). In contrast, mutation of 5 nt in the miR-203-3p seed sequence led to a complete abrogation of reporter inhibition (Fig 4B). We transfected miR-203-3p mimics or inhibitors into primary HSCs from uninfected mice, and quantified the level of IL-33 by qPCR or western blot. Our data revealed that, at both the mRNA and protein levels, elevation of miR-203-3p distinctly reduced the expression of IL-33, while depletion of miR-203-3p significantly increased the expression of IL-33 (Fig 4C and 4D). Finally, we analyzed IL-33 levels in primary HSCs from infected livers treated with rAAV8-pri-miR-203-3p, and found that IL-33 expression was markedly reduced (Fig 4E and 4F). Taken together, these data indicate that IL-33 is a direct target of miR-203-3p in HSCs. In addition, we noticed that the target site of miR-203-3p in the 3’UTR of *Il33* gene is not conserved from mouse to human. However, we provided evidence that human IL-33 is also a direct target of miR-203-3p in the HSCs (S5 Fig).

### MiR-203-3p targets IL-33 to regulate the expression of IL-13 in hepatic ILC2 cells during infection

**Fig 5:**
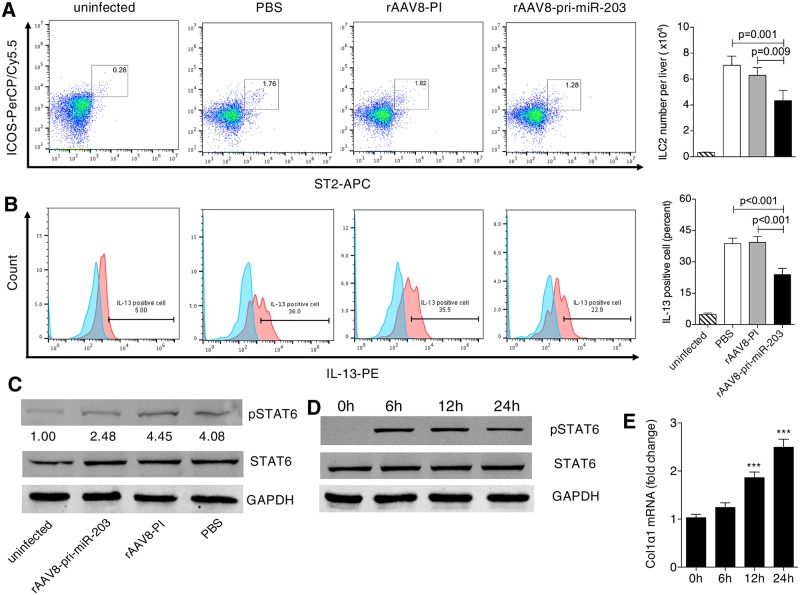
Production of IL-13 in hepatic ILC2s and STAT6 activation in HSCs after elevation of miR-203-3p in the infected livers. (A, B, C) Mice were infected percutaneously with 16 *S*. *japonicum* cercariae at day 0 or remained uninfected. Infected mice received rAAV8-PI or rAAV8-pri-miR-203-3p vectors at a dose of 1×10^11^ virus genomes or PBS by tail vein injection at day 10 post-infection. The number (A) and IL-13 production (B) of hepatic ILC2s were analyzed by flow cytometry, and activation of STAT6 pathway in HSCs (C) was detected by western blot at day 42 post-infection. Striped bars, uninfected mice (n = 4); white bars, mice receiving PBS (n = 4); grey bars, mice receiving rAAV8-PI (n = 4); black bars, mice receiving rAAV8-pri-miR-203-3p (n = 4). Data are expressed as the mean ± s.d. from two independent experiments. Multiple comparisons were performed by one-way ANOVA, and followed by Bonferroni post test for comparison between two groups. (D, E) Activation of STAT6 pathway (D) and collagen production (E) in HSCs after stimulation with IL-13. *** *P*<0.001, compared with control samples (0 h). Data are expressed as the mean ± s.d. from three independent experiments. Multiple comparisons were performed by one-way ANOVA, and followed by Bonferroni post test for comparison between two groups.

Though a number of cell types were suggested as sources of IL-13 in response to IL-33 stimulation, a recent study has proved that ILC2s, instead of lymphocytes, basophils, or mast cells, are the predominant source of IL-13 in the liver after IL-33 stimulation [19]. Having found that IL-33 is a target of miR-203-3p and that elevation of miR-203-3p leads to a reduction of IL-13 in the liver, we hypothesized that down-regulation of miR-203-3p during the progression of hepatic schistosomiasis could lead to higher expression of IL-13 in ILC2s via increased levels of IL-33 in HSCs. To address this, we first analyzed the number of hepatic ILC2s and production of IL-13 by hepatic ILC2s during infection using flow cytometry (S6 Fig). Our data indicated that ILC2s were a primary source of IL-13 production in the infected livers (S7 Fig), and both the number of ILC2s and production of IL-13 in these cells were increased by day 32 post-infection, peaking at day 42 post-infection (S8A Fig). This initial elevation occurred prior to the elevation of IL-33 in HSCs (Fig 3D and S8B Fig). STAT6 phosphorylation, a marker of IL-13 pathway activation, also began to increase in HSCs, peaking at the same time point, and the production of collagen in HSCs was dramatically elevated at day 42 post-infection (S8B Fig). Moreover, we observed that, following elevation of miR-203-3p in HSCs using rAAV8-pri-miR-203-3p vectors, the number of ILC2s and production of IL-13 in these cells were markedly reduced (Fig 5A and 5B). The expression of IL-33 (Fig 4E and 4F), the phosphorylation of STAT6 (Fig 5C), and production of collagen (S2 Fig) in HSCs were all significantly decreased. Finally, we found that purified primary HSCs, stimulated with recombinant IL-13 *ex vivo*, responded by phosphorylation of STAT6 and production of collagen in a time-dependent manner (Fig 5D and 5E). Taken together, these data indicate that miR-203-3p regulates the expression of IL-13 in ILC2s by targeting IL-33 in HSCs, thus modulating the expression of ECM by HSCs, during the progression of hepatic schistosomiasis.

## Discussion

In this study, we demonstrate that efficient and sustained elevation of miR-203-3p in liver tissues, using the highly hepatotropic rAAV8, protects mice against lethal schistosome infection by alleviating hepatic fibrosis. Importantly, we show that miR-203-3p targets IL-33, an inducer of type 2 immunity, in HSCs to regulate the expansion and IL-13 production of hepatic ILC2s during infection.

Type 2 immune response, featured by elevation of IL-4 and IL-13 levels, plays a crucial role in host protection as well as pathological tissue fibrosis after helminth infection, including schistosome infection, but the signals that induce type 2 immunity are poorly understood. Recently, numerous studies have highlighted that tissue damage, which induces the release of cytokine alarmins such as IL-33, is a potent mechanism driving type 2 immunity, particularly in the context of helminth infection [20,21]. The role of IL-33 in schistosomiasis has also been intensively studied, but published reports have been inconsistent. Mchedlidze *et al*. reported that, in the animal model of *Schistosoma mansoni* (*S*. *mansoni*) infection, IL-33 was critical for inducing the development of IL-13-dependent hepatic fibrosis [19]. This phenomenon was also observed in the animal model of *S*. *japonicum* infection [22]. However, a recent study showed that IL-33 needed to synergize with two other cytokine alarmins, thymic stromal lymphopoietin (TSLP) and IL-25, in the regulation of IL-4/IL-13-dependent inflammation or fibrosis after *S*. *mansoni* infection [23]. The inconsistency might be due to the difference of intervention time: when IL-33 is depleted in the embryo stage, the function of IL-33 might be compensated by other factors; but when IL-33 is inhibited during the progression of diseases, the role of IL-33 in disease progression become obvious. In this study, our data also indicated that IL-33 is crucial for inducing the progression of type 2 pathology after infection, as significant reductions in hepatic fibrosis and IL-13-producing ILC2s were observed upon down-regulation of IL-33 in the liver, which resulted from rAAV8-mediated elevation of miR-203-3p. These studies, including our current study, have established that IL-33 is a crucial mediator in the maintenance of type 2 pathology induced by schistosome infection.

Importantly, our study revealed other important aspects of the role of IL-33 in the regulation of type 2 pathology after infection. Although IL-33 is involved in the progression of many human diseases, little is known about the regulation of its expression. Our study, for the first time, revealed that IL-33 is regulated by miRNAs. IL-33 is a direct target of miR-203-3p in HSCs, and downregulation of miR-203-3p leads to elevated levels of IL-33 in HSCs, initiating type 2 pathology after infection. Previous studies have demonstrated that miR-155 can regulate the expansion and IL-13 production of ILC2 in the context of IL-33 [24], and miR-29a can regulate IL-33 effector function via targeting its decoy receptor sST2 [25]. Thus, miRNA could be an important regulator in the initiation of type 2 pathology. Activated HSCs are a major source of IL-33 in infected livers. Our findings confirmed that IL-33-producing cells are located in the periphery of egg granulomas where activated HSCs produce excess collagen, and that expression of IL-33 in primary HSCs is significantly elevated after infection. These findings are consistent with previous studies, which demonstrated that activated HSCs and pancreatic stellate cells (PSCs) are major sources of IL-33 in the fibrotic liver and pancreas, respectively, of both mice and human [26–28]. These studies suggest that HSCs or PSCs might be the sentinel cells in the organs, which detect the injury signals and promote wound healing response by releasing damage-associated molecular pattern such as IL-33. Our data showed that the initial elevation of IL-13 in hepatic ILC2 cells (day 32 post-infection) occurs prior to the elevation of IL-33 in whole livers and HSCs (day 42 post-infection). It is reported that cell death by necrosis or active necroptosis, instead of active secretion, might be the dominant mechanism by which IL-33 reaches the extracellular milieu [29]. Therefore, we speculate that the source of IL-33 that activates hepatic ILC2s at day 32 after infection derives from the necrosis or active necroptosis of HSCs.

**Fig 6:**
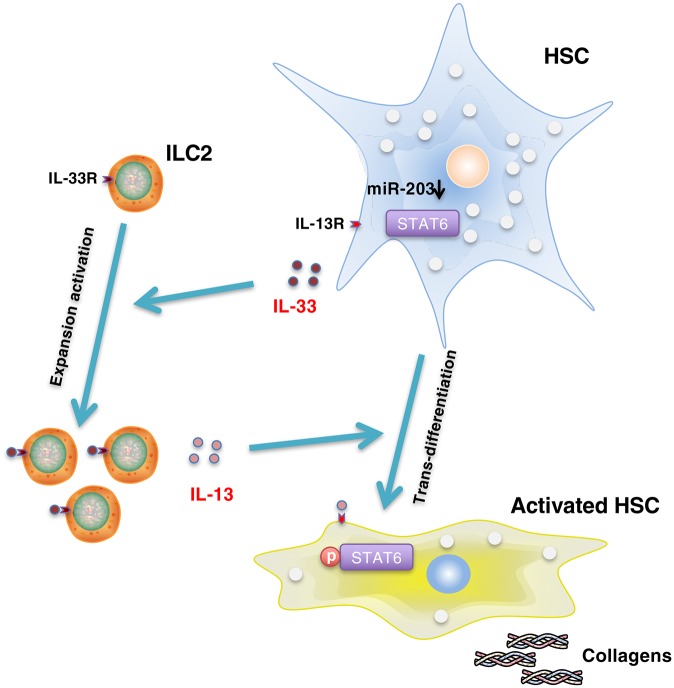
Schematic diagram of the role of miR-203-3p in the initiation of type 2 pathology after infection. Infection induces the down-regulation of miR-203-3p in HSCs, resulting in the elevation of IL-33. Extracellular IL-33 stimulates the expansion and IL-13 production of hepatic ILC2s. Elevated IL-13 then activates HSCs to produce excessive collagen through activation of the STAT6 pathway.

Here, we created a schematic diagram showing the molecular mechanism underpinning the regulation of type 2 pathology after infection by miR-203-3p (Fig 6): The toxic challenge derived from parasite eggs trapped in the liver tissue induces the down-regulation of miR-203-3p in HSCs, which relieves the inhibition to IL-33. Sequential elevation of IL-33 is released into the liver tissue and stimulates the proliferation and IL-13 production of hepatic ILC2s. IL-13 then activates HSCs to produce excessive ECMs through activation of STAT6 pathway. Thus, our study highlights the crucial role of miR-203-3p and its target IL-33 in the initiation of type 2 pathology during schistosome infection. It is noteworthy that IL-33 expresison in HSCs begins to elevate at day 42 after infection, thus, this mechanism mainly exerts its role in the progression of Th2 pathology after 42 days. In addition, IL-33 is reported to promote the development of fibrosis in many organs, including liver [19], lung [30], kidney [31], heart [32], skin [33], and other organs [34]. Therefore, miR-203-3p might serve as a useful target in the treatment of these fibrotic diseases.

## Materials and methods

### Ethics statement

All animal experiments were performed in strict accordance with the Guide for the Care and Use of Laboratory Animals of the National Institutes of Health, and were approved by the Animal Ethics Committee of Second Military Medical University (laboratory animal usage number FYXK (Shanghai) 2014–0003). To minimize pain and discomfort, all animal surgeries were undertaken under sodium pentobarbital anaesthesia.

### Mice and parasite infection

Male BALB/c mice (6 week) were obtained from the experimental animal center of Second Military Medicine University, and were housed under specific pathogen-free conditions and fed with autoclaved food and water as needed. To establish the animal model of schistosomiasis, mice were exposed percutaneously to 16 or 30 *S*. *japonicum* cercariae.

### Parasite perfusion, liver sample collection, and egg counting

For parasite perfusion, the portal vein was dissected at the root, then the thoracic cavity of the mouse was opened and the circulatory system was perfused via the aorta with sterile PBS. Parasites were collected and counted in a sterile petri dish containing medium. Subsequently, the liver was removed and snap-frozen in liquid nitrogen. For egg counting, part of the liver was digested overnight with 4% potassium hydroxide, then the total number of schistosome eggs was counted, and the liver egg burdens were defined as 10^4^ eggs per gram of liver tissue.

### Liver pathology measurement

The size of hepatic granuloma was measured from Mayer’s H&E staining of sections using a calibrated measuring eyepiece, and the extent of fibrosis was analysed by Masson’s trichrome staining of sections as described previously [15]. All granulomas within each section were scored for blue density on a scale of 1–4, and a second measurement of area involved was also determined using the same scale. The total fibrosis score was determined by multiplying the density and area for each granuloma (a score of 16 would be the maximum). The hydroxyproline content in the liver was detected using a colourmetric assay kit according to the manufacturer’s instructions (Nanjing Jiancheng Bioengineering Institute, Nanjing, China).

### Isolation of primary HSCs, hepatocytes, and Kupffer cells

The procedure was performed as described previously [15]. In short, HSCs were first isolated by density-gradient centrifugation and then further purified using negative selection with magnetic CD11b antibody beads (MACS, Miltenyi, Auburn, CA); Kupffer cells were first isolated by density-gradient centrifugation and then further purified using positive selection with magnetic CD11b antibody beads.

### Cell culture and treatment

Primary HSCs were cultured on plastic dishes in DMEM supplemented with 10% fetal bovine serum (Hyclone), 4 mmol/L L-glutamine, penicillin (100 IU/ml), and streptomycin (100 mg/ml). Cells were maintained at 37°C, 5% CO_2_ in a humidified atmosphere. For transfection, HSCs were transfected with 40 nM miR-203-3p mimics (Qiagen), miR-203-3p inhibitors (Qiagen), or negative controls at day 3 after seeding using Lipofectamine 3000 (Invitrogen).

### RNA extraction and real-time PCR

Total RNA was isolated using Trizol reagent (Invitrogen) according to the manufacturer’s protocol. Real-time PCR was performed as described previously [35]. The levels of miR-203-3p, *Col1α1*, *Col3α1*, *α-Sma*, *Ifn-γ*, *Tnf-α*, *Tgf-β1*, *Il4*, *Il5*, *Il10*, *Il13*, and *Il33* were detected using the SYBR Green Master Mix kit (Roche). U6 snRNA or *Gapdh* was used as an internal control, and the fold change was calculated by the 2^-ΔΔCt^ method. Sequences of primers used in this study are shown in S1 Table.

### Western blotting

Total cell protein was extracted on ice using RIPA lysis buffer in the presence of freshly added protease and phosphatase inhibitors (Thermo), then quantified by the BCA method (Pierce). A total of 30 μg protein extract per lane was loaded onto a 14% SDS-polyacrylamide gel and transferred to nitrocellulose membranes (Pierce). Nonspecific binding was blocked with 5% nonfat milk in PBS. The membrane was incubated with rat anti-IL-33 (R&D) or rabbit anti-phospho-STAT6 (Cell signaling) antibody overnight at 4°C. IRDye 800CW goat anti-rabbit IgG or goat anti-rat IgG (LI-COR) was used as secondary antibody, and rabbit anti-GAPDH antibody (Abcam) was used as an internal standard.

### Immunohistochemistry

Immunohistochemistry was performed on formaldehyde-fixed, paraffin-embedded mouse livers. After hydration, liver sections were incubated with rat anti-IL-33 (R&D) or rabbit anti-α-SMA (Abcam) antibody for 1 hour at 37°C, and HRP or fluorescence conjugated secondary antibody (Abcam) was used to display the signals.

### Flow cytometry

Nonparenchymal cells isolated from liver were stimulated with PMA (50 ng/mL), Ionomycin (1 μg/mL), and BFA (3 μg/mL) for 4 hours. Cells were surface stained with FITC conjugated lineage cocktail (CD3 / Gr-1 / CD11b / CD45R / Ter-119 / Siglec-f / CD11c / NK1.1) (Biolegend), PerCP/Cy5.5 conjugated ICOS (Biolegend) and APC conjugated ST2 (Biolegend), permeabilized with 0.1% spaonin buffer for 15 minutes, and further stained with PE conjugated IL-13 (eBioscience) before acquiring with FACS Calibur. ILC2s are defined as Lineage(-) ST2(+) ICOS(+) cells in this study. Data were analyzed in Flowjo.

### 3’UTR luciferase reporter constructs

The human or mouse wild-type or mutant 3’ UTRs of IL-33 containing the predicted miR-203-3p binding sites were synthesized (South Gene Technology, China) and cloned into the pGL3.0-control vectors according to the manufacturer’s instructions (Promega). 293T cells were seeded in 24-well plates, then transfected with 40 nM miR-203-3p or a negative control (Qiagen) and co-transfected with 0.8 μg per well wild-type IL-33 3’ UTR-luc or mutant IL-33 3’UTR-luc, using Lipofectamine 3000 (Invitrogen) according to the manufacturer’s instructions. pRL-TK vectors (0.1 μg per well) were co-transfected as endogenous controls for luciferase activity. After 24 h, cells were lysed, and luciferase activities were measured using a dual-luciferase assay kit (Promega).

### rAAV8 design and production

To express miR-203-3p, pri-miR-203-3p fragment was amplified by PCR from C57/B6 mouse genomic DNA using primer pri-miR-203-3pF (5´AACAGGTCCTCGCACAGAGTGCAGCCCGGC 3´) and pri-miR-203-3pR (5´AACAGGTCCTCCACCCCCGCGCCCCTCTCA3´), then cloned into the PpuMI restriction site in the intron of pscAAVCBPI GLuc plasmid [36]. The identity of pri-miR-203-3p was verified by sequencing. rAAV8 vectors used in this study were generated, purified, and tittered as described [37].

### Statistics

All analyses were carried out with the SPSS 19.0 software. Data were shown as mean ± s.d. The significance of difference between two groups was identified using a Student’s *t*-test. Multiple comparisons were performed by one-way ANOVA, and followed by Bonferroni post test for comparison between two groups. Survival between different groups was compared by Kaplan–Meier survival curves with log-rank test. *P* values less than 0.05 were considered significant.

## Supporting information

S1 Fig Alteration of cytokine expression, parasite burden, and virus delivery after the administration of rAVV8 vectors. (A) The expression of *Ifn-γ*, *Tnf-α*, *Il4*, and *Il5* mRNA in the liver was detected by qPCR. (B) The parasite living in the host and egg burden in the liver was counted. (C) Transduced vector genomes of the livers were detected by real-time PCR. (D) Gluc activities in the serum. The experiment design was described in Fig 1(B)–1(K).
(TIF)

S2 Fig MiR-203-3p regulates the activation of HSCs. Mice were infected percutaneously with 16 *S*. *japonicum* cercariae at day 0 or remained uninfected. Infected mice received rAAV8-PI or rAAV8-pri-miR-203-3p vectors at a dose of 1×10^11^ virus genomes or PBS by tail vein injection at day 10 post-infection. Primary HSCs were isolated at day 42 post-infection, then total RNA was collected and analyzed for expression of collagen 1, collagen 3, and *α-Sma* by qPCR. Striped bars, uninfected mice (n = 3); white bars, mice receiving PBS (n = 3); grey bars, mice receiving rAAV8-PI (n = 3); black bars, mice receiving rAAV8-pri-miR-203-3p (n = 3). Data are expressed as the mean ± s.d. from two independent experiments.
(TIF)

S3 Fig Immunochemistry staining for IL-33 and α-SMA in the infected (day 42 post-infection) livers. (TIF)

S4 Fig Levels of miR-203-3p, IL-33, and collagen in HSCs during *in vitro* activation were detected by qPCR. Primary HSCs from naive mice were isolated and cultured on a plastic plate. Cells were collected at various time points to detect the expression of *Il33* mRNA, *Col1α1* mRNA, and miR-203-3p using qPCR.
(TIF)

S5 Fig Relationship between miR-203-3p and IL-33 in human system. (A) Sequence alignment of miR-203-3p and its target sites in 3’ UTRs of human *Il33*. (B) Luciferase reporter assays for 293T cells transfected with pRL-TK vectors carrying human *Il33* wild type (WT) 3’ UTR or *Il33* mutant (Mut) 3’ UTR in the absence or presence of miR-203-3p mimics. (C) A human immortal HSC cell line, LX-2, was transfected with 40 nM miR-203-3p mimics, negative control (NC) miRNA mimics, miR-203-3p inhibitors, or negative control (NC) miRNA inhibitors for 48 h, then the expression of IL-33 was detected by western blot.
(TIF)

S6 Fig The gate strategy of flow cytometry in the detection of numbers of ILC2 cells and production of IL-13 by ILC2s. The lineage cocktail includes CD3, Gr-1, CD11b, CD45R, Ter-119, Siglec-F, CD11c, and NK1.1.
(TIF)

S7 Fig The IL-13 production in different cell subpopulations. Four mice were infected with 16 percutaneously with 16 *S*. *japonicum* cercariae at day 0, and sacrificed at day 42. The IL-13 production of different subpopulations in nonparenchymal cells, including Lin(+) cells, Lin(-) cells, Lin(-) ST(+) ICOS (+) cells, and Lin(-) ST(-) ICOS (-) cells, were analyzed by flow cytometry. (A) This figure shows the result of a presentative liver sample. (B) Percent of IL-13 positive cell in different cell subpopulations.
(TIF)

S8 Fig Activation of ILC2s and IL-13 pathway during infection. (A) The number of hepatic ILC2 cells and production of IL-13 by hepatic ILC2s during infection were analyzed using flow cytometry. (B) The expression of IL-33, phospho-STAT6, and collagen in HSCs during infection was analyzed by western blot or qPCR.
(TIF)

S1 Table Primer sequences used in the study. (PDF)
