# Supplementary figures and images for: Down-regulation of microRNA-203-3p initiates type 2 pathology during schistosome infection via elevation of interleukin-33
Source: PLoS Pathog. 2018 Mar 19;14(3):e1006957. doi: 10.1371/journal.ppat.1006957 (PMC5875897; doi:10.1371/journal.ppat.1006957)

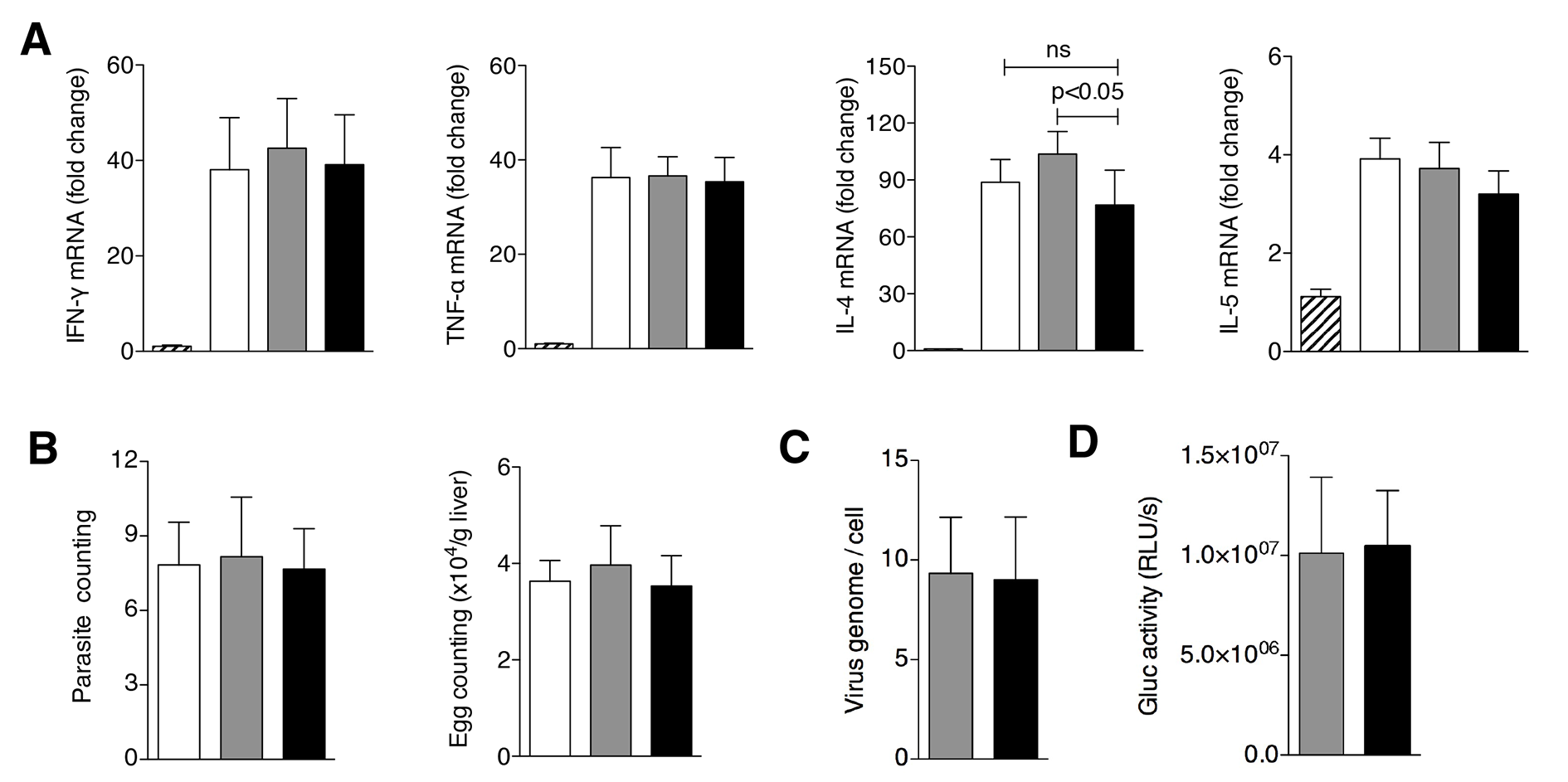

Supplement: S1 Fig — (A) The expression of Ifn-γ, Tnf-α, Il4, and Il5 mRNA in the liver was detected by qPCR. (B) The parasite living in the host and egg burden in the liver was counted. (C) Transduced vector genomes of the livers were detected by real-time PCR. (D) Gluc activities in the serum. The experiment design was described in Fig 1(B)–1(K). (TIF) [file ppat.1006957.s001.tif]

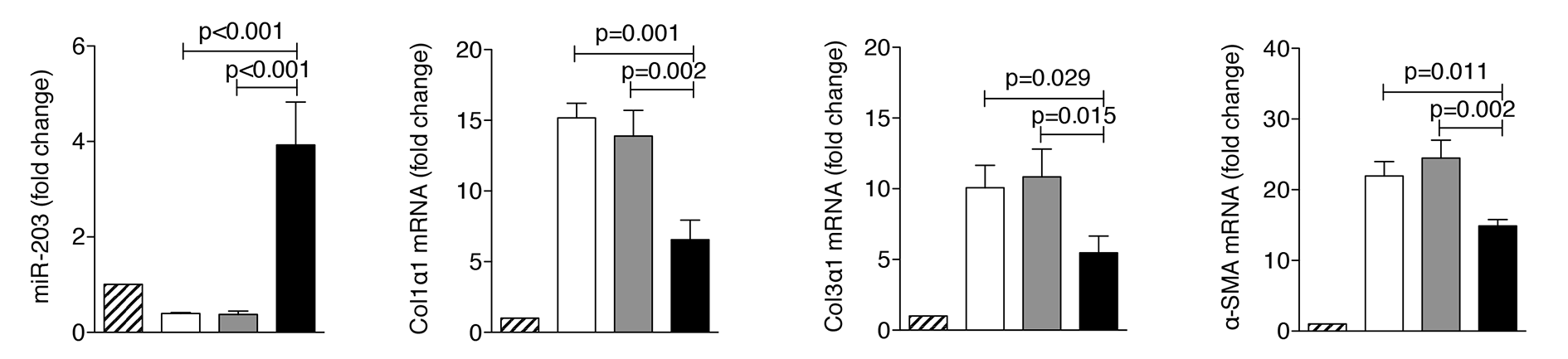

Supplement: S2 Fig — Mice were infected percutaneously with 16 S. japonicum cercariae at day 0 or remained uninfected. Infected mice received rAAV8-PI or rAAV8-pri-miR-203-3p vectors at a dose of 1×10^11^ virus genomes or PBS by tail vein injection at day 10 post-infection. Primary HSCs were isolated at day 42 post-infection, then total RNA was collected and analyzed for expression of collagen 1, collagen 3, and α-Sma by qPCR. Striped bars, uninfected mice (n = 3); white bars, mice receiving PBS (n = 3); grey bars, mice receiving rAAV8-PI (n = 3); black bars, mice receiving rAAV8-pri-miR-203-3p (n = 3). Data are expressed as the mean ± s.d. from two independent experiments. (TIF) [file ppat.1006957.s002.tif]

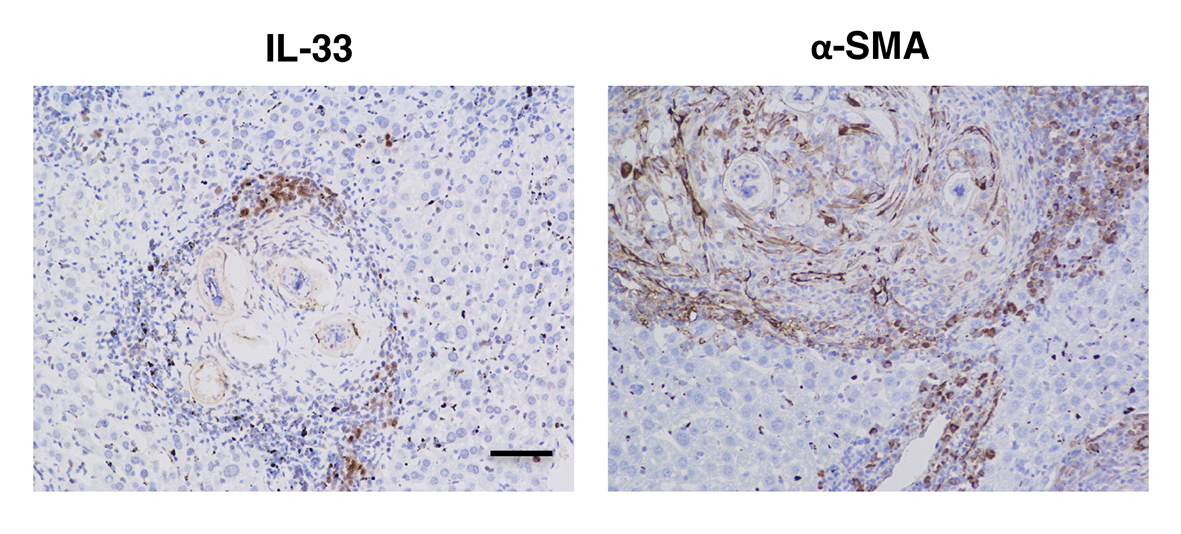

Supplement: S3 Fig — (TIF) [file ppat.1006957.s003.tif]

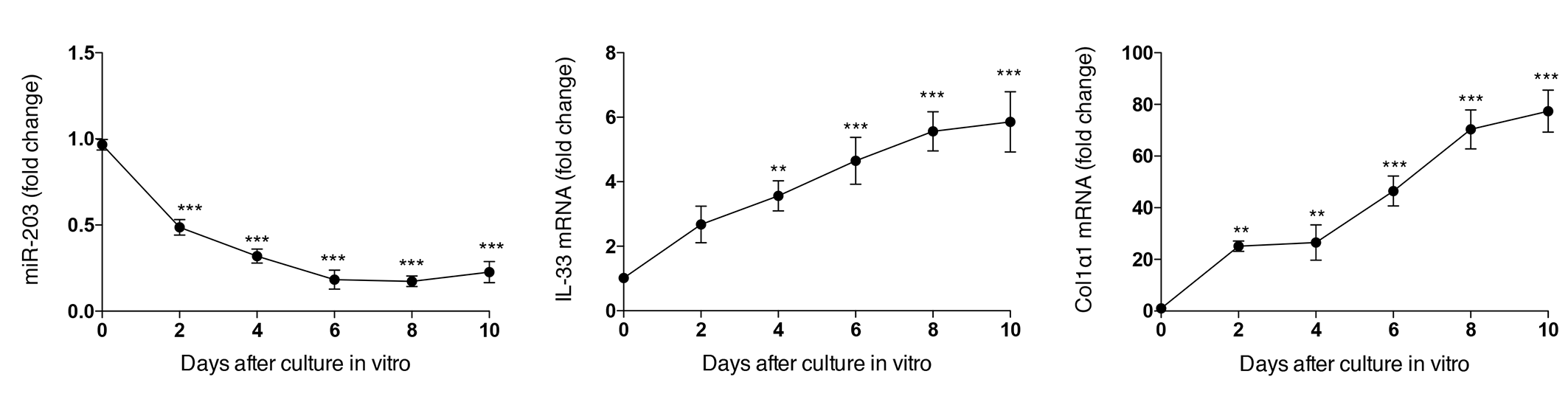

Supplement: S4 Fig — Primary HSCs from naive mice were isolated and cultured on a plastic plate. Cells were collected at various time points to detect the expression of Il33 mRNA, Col1α1 mRNA, and miR-203-3p using qPCR. (TIF) [file ppat.1006957.s004.tif]

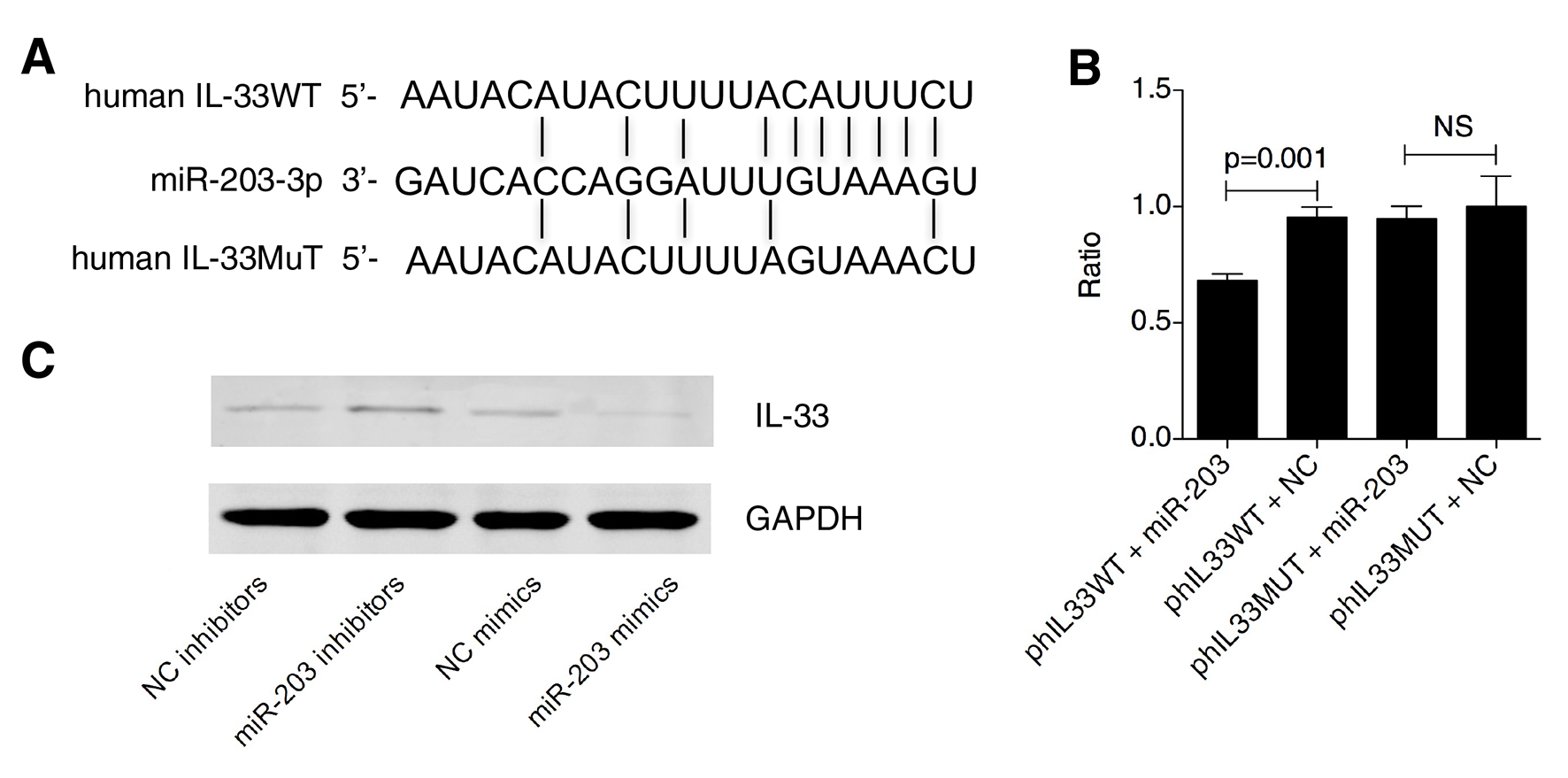

Supplement: S5 Fig — (A) Sequence alignment of miR-203-3p and its target sites in 3’ UTRs of human Il33. (B) Luciferase reporter assays for 293T cells transfected with pRL-TK vectors carrying human Il33 wild type (WT) 3’ UTR or Il33 mutant (Mut) 3’ UTR in the absence or presence of miR-203-3p mimics. (C) A human immortal HSC cell line, LX-2, was transfected with 40 nM miR-203-3p mimics, negative control (NC) miRNA mimics, miR-203-3p inhibitors, or negative control (NC) miRNA inhibitors for 48 h, then the expression of IL-33 was detected by western blot. (TIF) [file ppat.1006957.s005.tif]

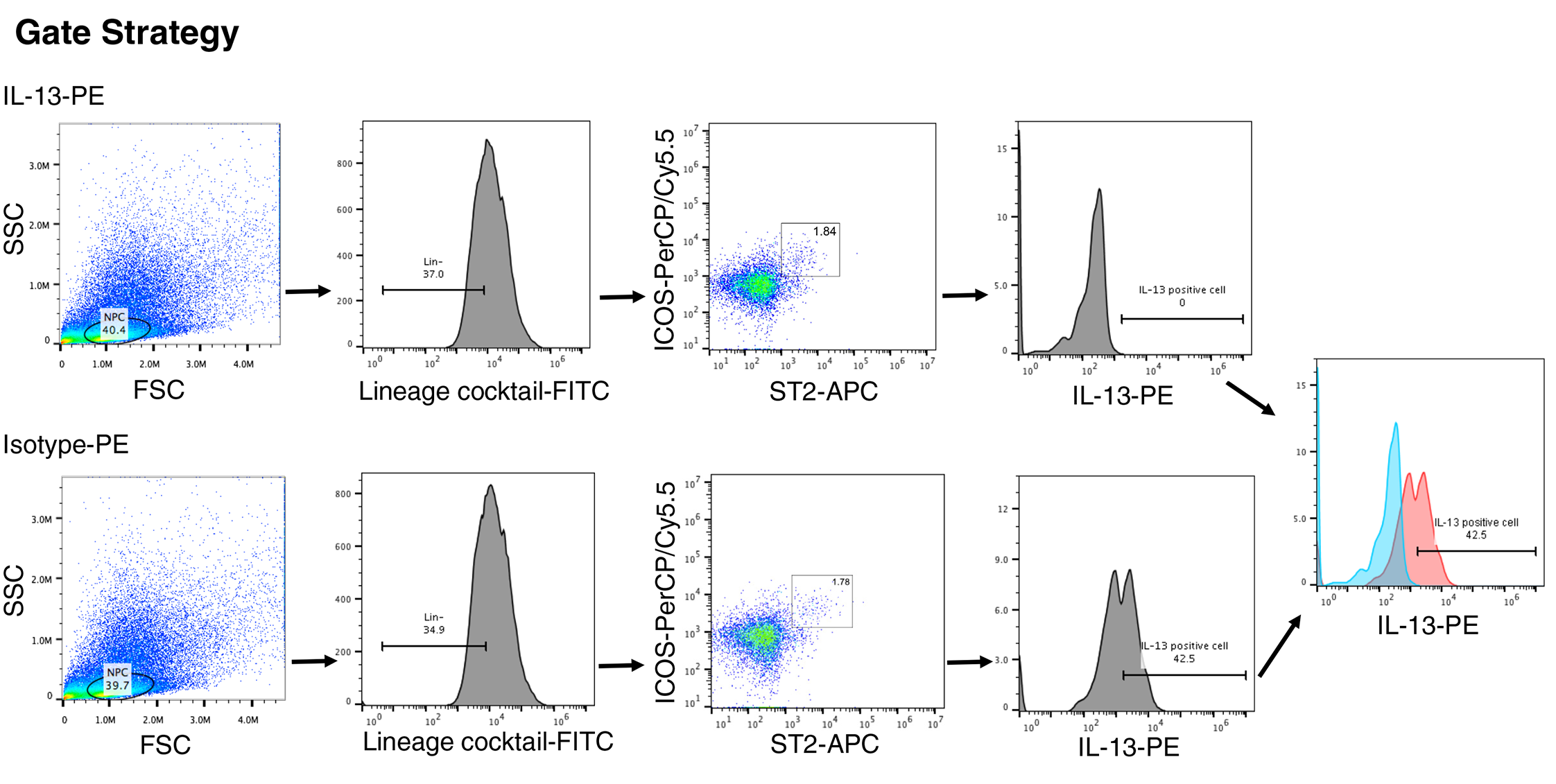

Supplement: S6 Fig — The lineage cocktail includes CD3, Gr-1, CD11b, CD45R, Ter-119, Siglec-F, CD11c, and NK1.1. (TIF) [file ppat.1006957.s006.tif]

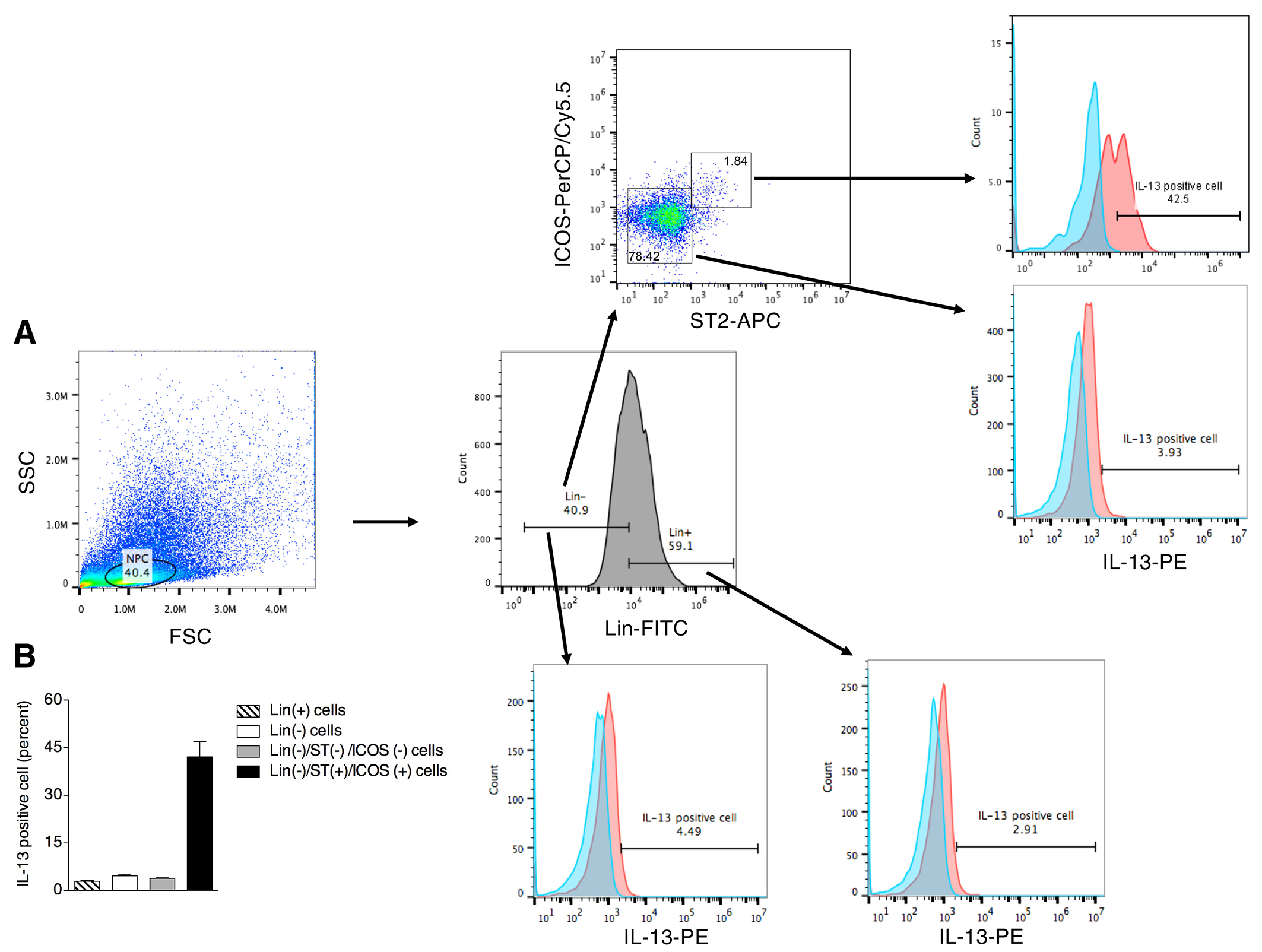

Supplement: S7 Fig — Four mice were infected with 16 percutaneously with 16 S. japonicum cercariae at day 0, and sacrificed at day 42. The IL-13 production of different subpopulations in nonparenchymal cells, including Lin(+) cells, Lin(-) cells, Lin(-) ST(+) ICOS (+) cells, and Lin(-) ST(-) ICOS (-) cells, were analyzed by flow cytometry. (A) This figure shows the result of a presentative liver sample. (B) Percent of IL-13 positive cell in different cell subpopulations. (TIF) [file ppat.1006957.s007.tif]

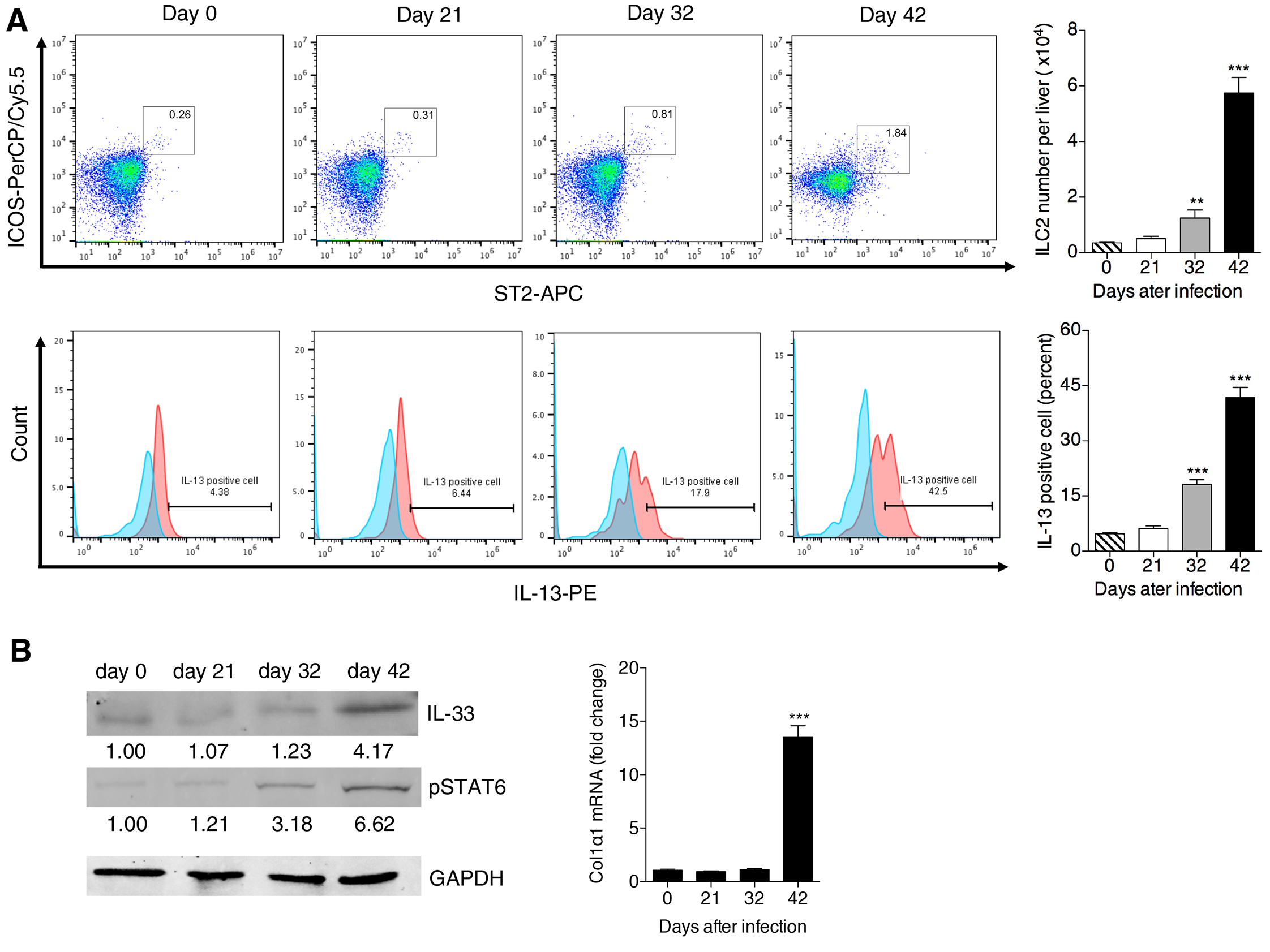

Supplement: S8 Fig — (A) The number of hepatic ILC2 cells and production of IL-13 by hepatic ILC2s during infection were analyzed using flow cytometry. (B) The expression of IL-33, phospho-STAT6, and collagen in HSCs during infection was analyzed by western blot or qPCR. (TIF) [file ppat.1006957.s008.tif]
