# Supplementary material for: Down-regulation of microRNA-203-3p initiates type 2 pathology during schistosome infection via elevation of interleukin-33
Source: PLoS Pathog. 2018 Mar 19;14(3):e1006957. doi: 10.1371/journal.ppat.1006957 (PMC5875897; doi:10.1371/journal.ppat.1006957)
Supplement: S1 Table — (PDF) [file ppat.1006957.s009.pdf]

gene		primer sequence (5'→3')
miR-203	RT stem-loop primer	GTCGTATCCAGTGCAGGGTCCGAGGTATTCGCACTGGATACGACCTAGTGGT
	forward primer	ATGGTTCGTGGGGTGAAATGTTTAGGAC
	reverse primer	GCAGGGTCCGAGGTATTC
U6	forward primer	GCTTCGGCAGCACATATACTAAAAT
	reverse primer	CGCTTCACGAATTTGCGTGTCAT
TGF-β1	forward primer	TGACGTCACTGGAGTTGTACGG
	reverse primer	GGTTCATGTCATGGATGGTGC
Col 1α1	forward primer	GCACGAGTCA CACCGGAAC
	reverse primer	CCAATGTCCAAGGGAGCCAC
Col 3α1	forward primer	TGGTCCTCAGGGTGTAAAGG
	reverse primer	GTCCAGCATCACCTTTTGGT
α-SMA	forward primer	CGCTGCTCCAGCTATGTGTGA
	reverse primer	TTTGGCCCATTCCAACCATTAC
IL-13	forward primer	GGAGCTGAGCAACATCACACA
	reverse primer	GGTCCTGTAGATGGCATTGCA
IL-4	forward primer	GGTCTCAACCCCCAGCTAGT
	reverse primer	GCCGATGATCTCTCTCAAGTGAT
IL-10	forward primer	GCTCTTACTGACTGGCATGAG
	reverse primer	CGCAGCTCTAGGAGCATGTG
IL-5	forward primer	GCAATGAGACGATGAGGCTTC
	reverse primer	GCCCCTGAAAGATTTCTCCAATG
IFN-γ	forward primer	ATGAACGCTACACACTGCATC
	reverse primer	CCATCCTTTTGCCAGTTCCTC
TNF-α	forward primer	ATCCGCGACGTGGAACTG
	reverse primer	ACCGCCTGGAGTTCTGGAA
IL-33	forward primer	ATTTCCCCGGCAAAGTTCAG
	reverse primer	AACGGAGTCTCATGCAGTAGA
GAPDH	forward primer	ACCACAGTCCATGCCATCAC
	reverse primer	TCCACCACCCTGTTGCTGTA
